# Length of stay in Denmark before HIV diagnosis and linkage to care: a population-based study of migrants living with HIV, Denmark, 1995 to 2020

**DOI:** 10.2807/1560-7917.ES.2022.27.30.2100809

**Published:** 2022-07-28

**Authors:** Olivia Borchmann, Lars Haukali Omland, Jan Gerstoft, Carsten Schade Larsen, Isik Somuncu Johansen, Suzanne Lunding, Janne Jensen, Niels Obel, Ann-Brit Eg Hansen

**Affiliations:** 1 Department of Infectious Diseases, Copenhagen University Hospital, Hvidovre Hospital, Hvidovre, Denmark; 2 Department of Infectious Diseases, Copenhagen University Hospital, Rigshospitalet, Copenhagen, Denmark; 3 Department of Infectious Diseases, Aarhus University Hospital, Aarhus, Denmark; 4 Department of Infectious Diseases, Odense University Hospital, Odense, Denmark; 5 Department of Internal Medicine, Herlev University Hospital, Herlev, Denmark; 6 Department of Internal Medicine, Kolding Hospital, Kolding, Denmark

**Keywords:** HIV, transients and migrants, delayed diagnosis, continuity of patient care, health services

## Abstract

**Background:**

Migrants face an increased risk of HIV infection and late presentation for HIV care.

**Aim:**

To examine delays in HIV diagnosis, linkage to care (LTC), and risk of late presentation for migrants living with HIV in Denmark.

**Methods:**

We conducted a population-based, nationwide study of adult migrants (n = 2,166) presenting for HIV care between 1 January 1995 and 31 December 2020 in Denmark. Time from immigration to HIV diagnosis and from diagnosis to LTC, and late presentation were assessed, stratified by migrants’ geographical regions of origin, using descriptive statistics.

**Results:**

The demographics of the migrant population changed over time. Overall, migrants diagnosed with HIV after immigration to Denmark resided a median of 3.7 (IQR: 0.8–10.2) years in Denmark before diagnosis. Median time from HIV diagnosis to LTC was 6 (IQR: 0–24) days. Migrants diagnosed with HIV infection before immigration had a median of 38 (IQR: 0–105) days from arrival in Denmark to LTC. The corresponding median times for 2015–20 alone were 4.1 (IQR: 0.9–13.1) years, 0 (IQR: 0–8) days, and 62 (IQR: 25–152) days, respectively. The overall proportion of late presentation among migrants diagnosed with HIV after immigration was 60%, and highest among migrants from sub-Saharan Africa and East and South Asia.

**Conclusion:**

HIV diagnosis is still substantially delayed in Danish migrants, while LTC is timely. The proportions with late presentation are high. These results call for targeted interventions to reduce the number of migrants with undiagnosed HIV infections and of late presenters.

## Introduction

Migrants in Europe are disproportionately affected by the HIV epidemic [1,2] and are recognised by the United Nations (UN) as one of the most vulnerable populations for HIV acquisition [1]. In Europe, migrants constitute an estimated 10% of the population, a proportion which is growing [3]. In Denmark, which has an estimated prevalence of HIV infection of 0.11%, migrants comprised 52% of incident cases presenting for HIV care in 2019 [4], whereas they constituted 10% of the Danish population [5]. Many migrants originate from countries with a high prevalence of HIV infection. Several other factors, which reflect the socioeconomic status of migrants before, during, and after migration, may also contribute to migrants’ excess vulnerability to HIV acquisition [1,6,7].

Migrants in Europe [2,8] and Denmark [9] face not only an increased risk of being infected with HIV, but also of presenting late for HIV care. Several studies have shown how barriers such as legal status and fear of legal repercussions, racism, and stigma may prevent migrants living with HIV (MLWH) from seeking timely HIV care [10-12]. Late presenters (LPs) face increased risk of adverse outcomes, including increased short-term mortality, related to their HIV infection [8,13] and miss the health benefits of the recommended early initiation of antiretroviral therapy (ART) [14]. Thus, the medical consequences of late presentation exacerbate inequality in healthcare. Late presentation also presents a challenge for public health efforts to use treatment as prevention of new HIV infections [15,16].

Timely HIV care starts with timely HIV diagnosis, as described in the HIV Continuum of Care [17]. A diagnosis of HIV infection, however, does not guarantee linkage to care (LTC). The HIV epidemic among MSM (men who have sex with men) in Denmark has been thoroughly mapped by previous research from the Danish HIV Cohort [18,19]. However, there is lack of information about the dynamics of the HIV epidemic and the HIV continuum of care among MLWH, and about how it has changed over the past 25 years, where there have been considerable changes in migration dynamics, policies, diagnostic practices, and access to care.

Internationally, little work has been done to study time from migration to diagnosis and LTC among MLWH [20,21]. Determining this is essential to understand how delays in the HIV continuum of care after immigration may contribute to late presentation among MLWH. Such knowledge could guide targeted interventions to reduce the number of undiagnosed and untreated patients with HIV infections.

We aimed to determine the time from immigration to diagnosis of HIV infection and from HIV diagnosis to LTC and to examine late presentation, stratified by migrant geographical region of origin and calendar period, in Denmark over a 25-year period.

## Methods

We conducted a nationwide, population-based, retrospective, observational study of all MLWH registered in the Danish HIV Cohort Study (DHCS) between 1995 and 2020.

### Setting and testing strategies

In Denmark, all HIV-infected individuals are referred to one of eight specialised HIV healthcare centres, where they are seen on outpatient visits at proposed 12 to 48-week intervals. HIV testing, care and ART are offered free of charge to all individuals living with HIV who have legal residence in Denmark. The Box shows an overview of the Danish HIV testing strategies [9,22].

Box **The four primary HIV testing strategies, Denmark, 1995–2020**

**General practice**

• Available for migrants with legal residence

• Recommended optional HIV test at first visit to GP for migrants from high HIV prevalence regions

• Recommended optional yearly HIV test for all MSM

• Routine optional pregnancy screening since 2010

• Symptom-based testing

**Hospital**

• Available for all migrants with legal residence and, in case of emergency treatment, also for undocumented migrants

• HIV test offered to individuals presenting with possibly HIV-related symptoms

• HIV test for undocumented migrants as part of emergency treatment

**Community-based clinics in the four largest cities**

• Available for migrants, including undocumented, from Africa, Asia, Latin America and Eastern Europe, and all migrants who are MSM or transgender since 2008

• Drop-in, anonymous HIV test

**Red Cross clinic/refugee camp**

• Available for asylum seekers/quota refugees

• Voluntary health assessments at Red Cross clinics for asylum seekers (do not routinely include HIV test)

• Mandatory health assessments by IOM in refugee camps abroad for quota refugees (include routine optional HIV test)

GP: general practice; IOM: International Organization for Migration; MSM: men who have sex with men.

### Data sources

The DHCS, described in detail elsewhere [23], is a nationwide, population-based cohort study of all individuals living with HIV treated at Danish HIV healthcare centres since 1 January 1995. Individuals are continuously enrolled. Data including demographics, date of HIV diagnosis, first contact with a Danish HIV centre, AIDS-defining events, and ART are updated yearly.

The Danish Civil Registry System (CRS), which contains demographic data and vital status on all Danish residents, was accessed to obtain information on immigration and emigration dates and countries of origin. The Danish National Hospital Registry (DNHR), containing information on all non-psychiatric hospital admissions since 1977 and all outpatient visits since 1995, was used to obtain information on dates of first visits to specialised infectious diseases departments. Data were linked through the unique personal identification numbers given to all residents in Denmark.

### Study population

We included all individuals with HIV-1 in DHCS who (i) immigrated to Denmark and obtained residence, (ii) were aged 16 years or older at time of first contact with an HIV healthcare centre and (iii) had first contact with a Danish HIV healthcare centre between 1 January 1995 and 31 December 2020. Undocumented migrants are not registered in the CRS and were not included.

### Definitions

Data on country of origin of MLWH were obtained from the CRS. We categorised MLWH into five geographical regions of origin based on the United Nations Standard country and Area codes classifications [24]: (i) sub-Saharan Africa (SSA), (ii) western countries including northern, southern, and western Europe, United States (US) and Canada, and Australia and New Zealand, (iii) East and South Asia, (iv) eastern Europe and (v) other (North Africa, the Middle East, Latin America, the Caribbean and MLWH with missing information on country of origin) (Supplementary Material: Appendix 1. Country codes). 

The date of HIV diagnosis was obtained from DHCS. It was defined as the date of the first positive HIV test, either in Denmark or abroad, as self-reported by MLWH. LTC was defined as date of first contact with a Danish HIV healthcare centre. This date was identified as either (i) date of first HIV RNA viral load (VL) measurement, (ii) date of first CD4^+^ T-cell measurement, (iii) date of ART initiation if ART was initiated in Denmark or (iv) date of first visit to a Danish specialised department of infectious diseases after the first positive HIV test and/or with HIV infection as the primary diagnosis as registered in the DNHR, whichever came first. If a measurement of VL or CD4^+^ T-cell count was conducted within 90 days after LTC, it was counted as a measurement of VL/CD4^+^ T-cells at time of LTC.

Migrants’ periods of residence in Denmark were defined by dates of immigration and subsequent dates of emigration obtained from the CRS. If the diagnosis of HIV infection occurred while the MLWH was residing in Denmark, the MLWH was categorised as diagnosed with HIV after immigration. If the diagnosis of HIV infection occurred while the MLWH was residing abroad, the MLWH was categorised as diagnosed with HIV before immigration.

According to the European consensus definition [25], late presentation (LP) was defined as presenting for HIV care with a CD4^+^ T-cell count < 350 cells/µl or presenting with an AIDS-defining event regardless of CD4^+^ T-cell count. Presentation with advanced HIV disease (AHD) was defined as presenting with a CD4^+^ T-cell count < 200 cells/µl or with an AIDS-defining event regardless of CD4^+^ T-cell count [25]. Presenting with AIDS was defined as having developed an AIDS-defining event no later than 30 days after LTC. Viral suppression was defined as a VL < 200 copies/ml and was used as a marker for being on ART.

### Outcomes

For MLWH diagnosed with HIV infection after immigration to Denmark, we examined time from immigration to HIV diagnosis and time from HIV diagnosis to LTC. For MLWH diagnosed before immigration, we examined time from the first date of immigration following HIV diagnosis to LTC. We examined the proportions of LP and AHD.

### Statistics

All results were stratified according to MLWHs’ status as either diagnosed after or before immigration to Denmark.

MLWH could have several migrations in and out of Denmark before HIV diagnosis or LTC, and thus more than one period of residence in Denmark. Only time spent residing in Denmark contributed time to the outcomes.

We stratified our analyses according to calendar period and region of origin. The calendar periods were (i) 1 January 1995–31 December 1999, (ii) 1 January 2000–31 December 2004, (iii) 1 January 2005–31 December 2009, (iv) 1 January 2010–31 December 2014 (v) 1 January 2015–31 December 2020, as defined by the date of LTC. We chose 5-year periods to balance between having a sufficient number of individuals in each calendar period, while at the same time having enough calendar periods to be able to show possible trends over time. We conducted subanalyses restricted to LPs and MLWH with AHD and to MLWH from SSA and East and South Asia, stratified by calendar period. We performed a trend analysis using logistic regression to evaluate the developments over time in percentages of LP. 

A preliminary analysis revealed that a small proportion (n = 212) of MLWH diagnosed before immigration were registered with a date of immigration to Denmark, which occurred after linkage to Danish HIV care. They were assumed to be MLWH living in Denmark and receiving Danish HIV care before obtaining residence. In the calculations, their time from immigration to LTC was defined as 0 days.

A small number of MLWH had missing data on VL (n = 264) or CD4^+^ T-cell (n = 192) measurements. They were excluded from the analyses of VL, CD4^+^ T-cells and LP/AHD.

All calculations were done using descriptive statistics. SPSS statistical software version 25 was used for data analysis (IBM).

## Results

We identified 2,166 MLWH who fulfilled the inclusion criteria. Of these, 1,191 (55%) were men, and the median age was 33 (IQR: 28–40). Overall, 1,514 MLWH (70%) were diagnosed with HIV after immigration to Denmark. Of these, 202 (13%) had multiple periods of residence in Denmark before diagnosis. Of all MLWH, 652 (30%) were diagnosed with HIV before immigration. Over time, the number of MLWH diagnosed after immigration was constant, while the number diagnosed before immigration increased. Thus, the percentage of MLWH diagnosed before immigration to Denmark increased from 22% in 1995–99 to 41% in 2015–20. Characteristics of the two study populations are presented, stratified by region of origin (Table 1) and by calendar period (Table 2).

**Table 1:** Characteristics of migrants living with HIV, diagnosed before or after immigration, stratified by regions of origin, Denmark, 1995–2020 (n = 2,166)

Characteristics		Region of origin											
		All n = 2,166		Sub-Saharan Africa n = 891		Western countries n = 446		East and South Asia n = 403		Eastern Europe n = 171		Other n = 255	
		n	%	n	%	n	%	n	%	n	%	n	%
**Diagnosed after immigration**		1,514	70	655	74	250	56	293	73	128	75	188	74
Age in years, median (IQR)		33 (28–40)		32 (28–38)		37 (31–43)		34 (28–40)		31 (27–37)		34 (28–41)	
Sex^a^	Males	830	55	214	33	219	88	152	52	89	70	156	83
	Females	682	45	440	67	31	12	141	48	39	30	31	16
Reported HIV transmission modes	Sex between men	388	26	16	2	151	60	85	29	54	42	82	44
	Sex between men and women	873	58	551	84	62	25	156	53	41	32	63	34
	Injecting drug use	50	3	5	1	16	6	8	3	9	7	12	6
	Other/unknown	203	13	83	13	21	8	44	15	24	19	31	16
HIV-RNA values^b^	Measurement at LTC	1,335	88	562	86	215	86	277	95	115	90	166	88
	HIV-RNA (log_10_ copies/ml), median (IQR)	4.7 (3.9–5.2)		4.5 (3.7–5.2)		4.8 (4.0–5.4)		4.8 (4.0–5.3)		4.6 (4.0–5.3)		4.7 (4.1–5.2)	
	HIV-RNA < 200 copies/ml	78	6	42	7	15	7	13	5	≤ 4	≤ 3	≤ 4	≤ 2
CD4^+^ T-cell counts^b^	Measurement at LTC	1,421	94	615	94	229	92	283	97	120	94	174	93
	CD4^+^ T-cell count (cells/µL), median (IQR)^c^	290 (130–460)		250 (120–410)		362 (170–557)		276 (75–439)		341 (189–524)		341 (177–510)	
Late-stage HIV	Late presenters^d^	856	60	416	68	107	47	181	64	63	53	89	51
	Advanced HIV disease^e^	530	37	259	42	66	29	123	43	33	28	49	28
	AIDS	175	12	74	11	21	8	55	19	8	6	17	9
**Diagnosed before immigration**		652	30	236	26	196	44	110	27	43	25	67	26
Age in years, median (IQR)		33 (28–40)		33 (28–38)		36 (31–46)		31 (27–37)		30 (26–38)		32 (26–41)	
Sex	Males	361	55	70	30	165	84	47	43	25	NA	54	81
	Females	291	45	166	70	31	16	63	57	18	NA	13	19
Reported HIV transmission modes	Sex between men	202	31	10	4	114	58	26	24	16	NA	36	54
	Sex between men and women	279	43	172	73	29	15	55	50	12	NA	11	16
	Injecting drug use	20	3	≤ 4	≤ 2	9	5	≤ 4	≤ 4	5	NA	≤ 4	≤ 6
	Other/unknown	151	23	52	22	44	22	26	24	10	NA	19	28
HIV-RNA values^f^	Measurement at LTC	567	87	199	84	174	89	98	89	39	NA	57	85
	HIV-RNA (log_10_ copies/ml), median (IQR)	2.1 (1.3–4.4)		3.6 (1.3–4.7)		1.3 (1.3–3.5)		2.7 (1.3–4.7)		1.4 (1.3–4.0)		1.6 (1.3–3.5)	
	HIV-RNA < 200 copies/ml	296	52	68	34	119	68	45	46	25	NA	39	68
CD4^+^ T-cell counts^f^	Measurement at LTC	553	85	200	85	164	84	99	90	34	NA	56	84
	CD4^+^ T-cell count (cells/µL), median (IQR)^c^	430 (250–630)		311 (160–462)		525 (350–770)		440 (260–610)		550 (373–757)		521 (361–670)	
Late-stage HIV	CD4^+^ T-cell < 350 cells/µL/AIDS^f^	218	39	118	59	44	27	35	35	8	NA	13	23
	CD4^+^ T-cell < 200 cells/µL/AIDS^f^	122	22	70	35	22	13	21	21	≤ 4	NA	5	9
	AIDS	48	7	24	10	11	6	9	8	≤ 4	NA	≤ 4	≤ 6

AIDS: acquired immunodeficiency syndrome; IQR: interquartile range; LTC: linkage to care; NA: not applicable.

All characteristics were collected at date of LTC.

^a^ Missing information on sex was ≤ 4 (≤ 0.2%) in SSA and in Other.

^b^ Data were missing for some MLWH for the following variables: HIV-RNA (n = 179) and CD4^+^ T-cell count (n = 93) and were excluded from analyses of HIV-RNA, CD4^+^ T-cell count and late presentation/advanced HIV disease.

^c^ CD4^+^ T-cell normal range: 500–1,500 cells/µl.

^d^ Presenting with CD4^+^ T-cell count < 350 cells/µl or with an AIDS-defining event regardless of CD4^+^ T-cell count.

^e^ Presenting with CD4^+^ T-cell count < 200 cells/µl or with an AIDS-defining event regardless of CD4^+^ T-cell count.

^f^ Data were missing for some MLWH for the following variables: HIV-RNA (n = 85) and CD4^+^ T-cell count (n = 99) and were excluded from analyses of HIV-RNA and CD4^+^ T-cell count.

**Table 2:** Characteristics of migrants living with HIV, diagnosed before or after immigration, stratified by calendar periods, Denmark, 1995–2020 (n = 2,166)

Characteristics		Calendar period									
		1995–99 n = 394		2000–04 n = 414		2005–09 n = 426		2010–14 n = 456		2015–20 n = 476	
		n	%	n	%	n	%	n	%	n	%
**Diagnosed after immigration (n = 1,514)**		308	78	316	76	317	74	291	64	282	59
Age in years, median (IQR)		33 (28–38)		32 (29 – 38)		35 (28–41)		35 (30–41)		33 (28–41)	
Sex^a^	Males	154	50	150	47	164	52	170	58	192	68
	Females	154	50	166	53	153	48	121	42	88	31
Region of origin	Sub-Saharan Africa	161	52	169	53	137	43	108	37	80	28
	Western Countries	57	19	46	15	46	15	55	19	46	16
	East and South Asia	45	15	63	20	71	22	58	20	56	20
	Eastern Europe	5	2	12	4	27	9	36	12	48	17
	Other	40	13	26	8	36	11	34	12	52	18
Reported HIV transmission modes	Sex between men	54	18	54	17	76	24	92	32	112	40
	Sex between men and women	215	70	224	71	199	63	154	53	81	29
	Injecting drug use	11	4	17	5	9	3	10	3	≤ 4	≤ 1
	Other/unknown	28	9	21	7	33	10	35	12	86	30
HIV-RNA values^b^	HIV-RNA measurement at LTC	185	60	306	97	294	93	284	98	266	94
	HIV-RNA (log_10_ copies/ml), median (IQR)	4.6 (3.9–5.2)		4.7 (4.1 – 5.3)		4.5 (3.7–5.1)		4.7 (4.0–5.3)		4.7 (3.8–5.3)	
	HIV-RNA < 200 copies/ml	5	3	12	4	13	4	19	7	29	11
CD4^+^ T-cell counts^b^	CD4^+^ T-cell count at LTC	294	95	307	97	289	91	285	98	246	87
	CD4^+^ T-cell count (cells/µL), median (IQR)^c^	253 (95–400)		220 (89–440)		310 (163–441)		334 (152–524)		330 (179–502)	
Late-stage HIV	Late presenters^d^	197	67	205	67	168	58	151	53	135	55
	Advanced HIV disease^e^	124	42	147	48	91	31	98	34	70	28
	AIDS	45	15	47	15	38	12	33	11	12	12
**Diagnosed before immigration (n = 652)**		86	22	98	24	109	26	165	36	194	41
Age in years, median (IQR)		32 (28–38)		31 (26–37)		35 (30–41)		34 (28–41)		33 (29–41)	
Sex	Males	39	45	36	37	62	57	101	61	123	63
	Females	47	55	62	63	47	43	64	39	71	37
Region of origin	Sub-Saharan Africa	46	53	48	49	43	39	55	33	44	23
	Western Countries	21	24	19	19	34	31	51	31	71	37
	East and South Asia	15	17	24	24	14	13	28	17	29	15
	Eastern Europe	≤ 4	≤ 5	≤ 4	≤ 4	5	5	17	10	19	10
	Other	≤ 4	≤ 5	6	6	13	12	14	8	31	16
Reported HIV transmission modes	Sex between men	20	23	18	18	41	38	63	38	60	31
	Sex between men and women	51	59	69	70	61	56	56	34	42	22
	Injecting drug use	≤ 4	≤ 5	≤ 4	≤ 4	5	5	7	4	≤ 4	≤ 2
	Other/unknown	11	13	7	7	≤ 4	≤ 4	39	24	92	47
HIV-RNA values^f^	HIV-RNA measurement at LTC	53	62	85	87	97	89	151	92	181	93
	HIV-RNA (log_10_ copies/ml), median (IQR)	4.3 (2.8–5.1)		4.4 (3.4–5.0)		2.6 (1.6–4.3)		1.9 (1.3–4.4)		1.3 (1.3–1.5)	
	HIV-RNA < 200 copies/ml	6	11	10	12	45	46	84	56	151	83
CD4^+^ T-cell counts^f^	CD4^+^ T-cell count at LTC	74	86	93	94	93	85	148	90	145	75
	CD4^+^ T-cell count (cells/µL), median (IQR)^c^	270 (133–488)		262 (128–472)		376 (224–560)	435 (290–603)			600 (446–774)	
Late-stage HIV	CD4^+^ T-cell < 350 cells/µL/AIDS^f^	44	59	57	61	40	43	58	39	19	13
	CD4^+^ T-cell < 200 cells/µL/AIDS^f^	28	38	35	38	21	23	30	20	8	6
	AIDS	10	12	14	14	6	6	12	7	6	3

AIDS: acquired immunodeficiency syndrome; IQR: interquartile range; LTC: linkage to care; NA: not applicable.

All characteristics were collected at date of LTC.

^a^ Missing information on sex on ≤ 4 (≤ 0.2%) in 2015–20.

^b^ Data were missing for some MLWH for the following variables: HIV-RNA (n = 179) and CD4^+^ T-cell count (n = 93) and were excluded from analyses of HIV-RNA, CD4^+^ T-cell count and late presentation/advanced HIV disease.

^c^ CD4^+^ T-cell normal range: 500–1,500 cells/µl.

^d^ Presenting with CD4^+^ T-cell count < 350 cells/µl or with an AIDS-defining event regardless of CD4^+^ T-cell count.

^e^ Presenting with CD4^+^ T-cell count < 200 cells/µl or with an AIDS-defining event regardless of CD4^+^ T-cell count.

^f^ Data were missing for some MLWH for the following variables: HIV-RNA (n = 85) and CD4^+^ T-cell count (n = 99) and were excluded from analyses of HIV-RNA and CD4^+^ T-cell count.

### Migrants diagnosed with HIV infection after immigration

Among MLWH diagnosed with HIV after immigration, 830/1,514 (55%) were men and the median age at LTC was 33 (IQR: 28–40) years (Table 1). The demographic and HIV-related characteristics changed over time. From 1995–99, MLWH migrated predominantly from SSA (52%), 50% were women, and a high proportion (70%) reported heterosexual transmission. Over time, the regions of origin became more diverse, with an increasing proportion of MLWH from eastern Europe. More MLWH were men (68%) and MSM was more frequently reported as the route of infection (40%) (Table 2). In general, MLWH originating from SSA and East and South Asia were more frequently women and heterosexually infected and had lower CD4^+^ T-cell counts at LTC (< 300 cells/uL), whereas MLWH from Western countries and eastern Europe were predominantly men and MSM and had higher CD4^+^ T-cell (> 300 cells/uL) counts at LTC (Table 1).

#### Time from immigration to HIV diagnosis and from HIV diagnosis to LTC

**Figure 1:**
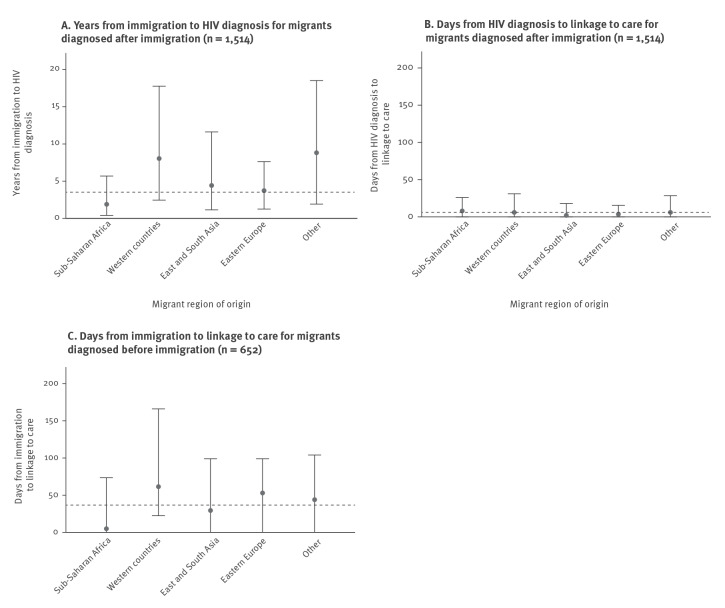
A. Time to HIV diagnosis and time to linkage to care for B. migrants diagnosed with HIV after immigration and C. migrants diagnosed with HIV before immigration^a^ stratified by migrant region of origin, Denmark, 1995–2020 (n = 2,166)

^a^ A total of 212 migrants were registered with a date of immigration which occurred after date of linkage to Danish HIV care. Their time from immigration to linkage to care was defined as 0 days.

The median time from immigration to HIV diagnosis was 3.7 (IQR: 0.8–10.2) years. There were notable geographical differences: time from immigration to HIV diagnosis was 1.9 (IQR: 0.4–5.7) years for MLWH from SSA and 8.0 (IQR: 2.5–17.8) years for MLWH from Western countries (Figure 1).

Graphs show median time (points), overall median (dashed line) and interquartile range (whiskers). Migrants’ region of origin was defined by United Nations standard country and area codes classifications [24].

**Figure 2:**
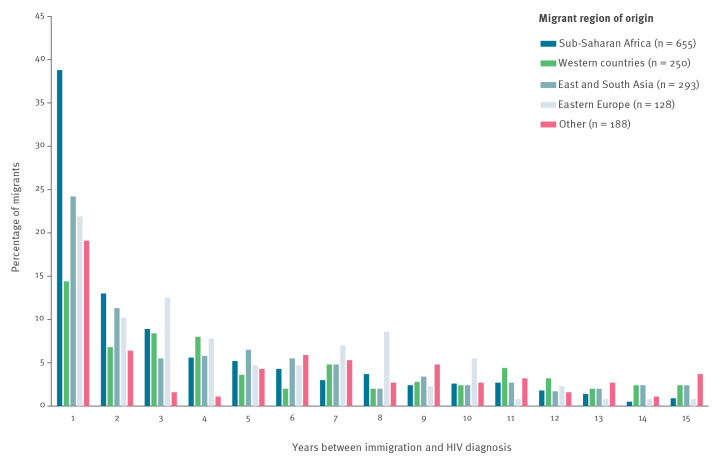
Distribution of migrants’ length of stay in years before HIV diagnosis^a^, Denmark, 1995–2020 (n = 1,514)

^a^ MLWH with > 15 years between migration and HIV diagnosis (n = 237) are not shown in the figure, but they were included in all calculations.

The proportion of MLWH diagnosed with HIV less than 1 year after immigration to Denmark ranged from 39% among MLWH from SSA to 14% among MLWH from Western countries (Figure 2).

**Figure 3:**
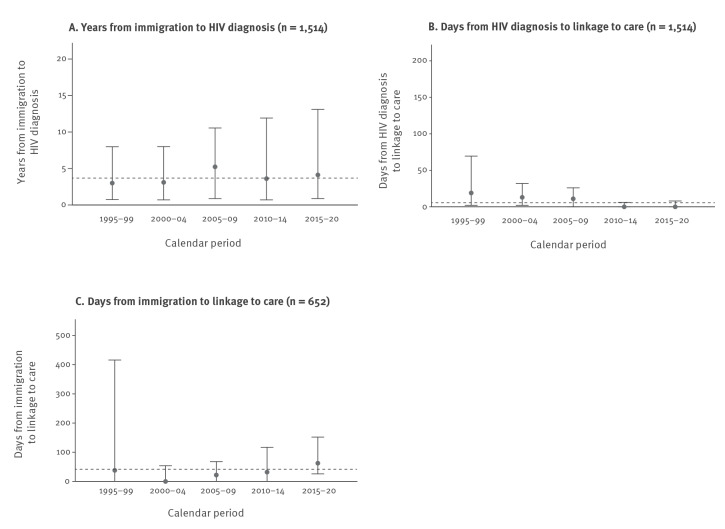
A. Time to HIV diagnosis and time to linkage to care for B. migrants diagnosed with HIV after immigration and C. migrants diagnosed with HIV before immigration^a^, stratified by calendar period, Denmark, 1995–2020 (n = 2,166)

^a^ A total of 212 migrants were registered with a date of immigration which occurred after date of linkage to Danish HIV care. Their time from immigration to linkage to care was defined as 0 days.

For the total population (Figure 3), as well as for MLWH from SSA and East and South Asia specifically (Supplementary Figure S1: Years (median, IQR) from immigration to HIV-diagnosis for MLWH from Sub-Saharan Africa and East and South Asia stratified by calendar period), time from immigration to HIV diagnosis was stable throughout the study period. In all analyses, we found a greater range in the duration of time from immigration to HIV diagnosis in the later calendar periods (Figure 3A).

Graphs show median time (points), overall median (dashed line) and interquartile range (whiskers). Migrants’ region of origin as defined by United Nations standard country and area codes classifications [24].

For all MLWH diagnosed after immigration, the median time from HIV diagnosis to LTC was 6 (IQR: 0–24) days. Between 223/250 (89%) from Western countries and 278/293 (95%) from East and South Asia and 122/128 (95%) from eastern Europe were linked to care within 90 days of HIV diagnosis. Time from HIV diagnosis to LTC was overall shortest in 2010–20 (Figure 3B).

#### Late presentation

MLWH from SSA and East and South Asia had the highest proportions of LP (68% and 64%, respectively) (Table 1). MLWH from East and South Asia had the highest proportion of AIDS at LTC (19%). Over the studied time, we observed a decline in the number and percentages of LP and AHD for the total MLWH population. In 1995–99, 197/294 (67%) were LPs and 124/294 (42%) had AHD compared with 135/246 (55%) and 70/246 (28%) respectively in 2015–20 (Table 2). However, the proportions of LP and AHD among MLWH from SSA and East and South Asia remained high over time. Among MLWH from SSA, 106 (69%) were LPs and 67 (44%) had AHD in 1995–99 compared with 42 (60%) and 25 (36%) in 2015–20, respectively. There was a slight but significant downwards trend in the percentage of LP (p value = 0.02). Among MLWH from East and South Asia, there was no significant change in the percentage of LP over time (p value = 0.45). In 1995–99, 33 (73%) were LPs and 18 (40%) had AHD compared with 36 (71%) LPs and 22 (43%) with AHD in 2015–20. (Supplementary Table S1: Late presentation and presentation with advanced HIV disease among migrants from Sub-Saharan Africa and East and South Asia stratified by calendar periods). 

Among women with available data on CD4^+^ T-cell counts (n = 646), 442/646 (68%) were LPs and 272/646 (42%) had AHD compared with 413/773 (53%) LPs and 258/773 (33%) with AHD among men with available data on CD4^+^ T-cell counts (n = 773). In 2015–20 among women, 50/76 (66%) were LPs and 28/76 (37%) had AHD compared with 85/168 (51%) LPs and 42/168 (25%) with AHD among men.

#### Time from immigration to HIV diagnosis for late presenters and migrants presenting with advanced HIV disease

For LPs and MLWH with AHD, the median times from immigration to HIV diagnosis were 3.5 (IQR: 0.9–9.3) and 3.9 (IQR: 1.0–9.4) years. Time from immigration to HIV diagnosis was stable over calendar time (Supplementary Figure S2: Years (median, IQR) from immigration to HIV-diagnosis for late presenters, stratified by calendar period).

### Migrants diagnosed with HIV infection before immigration

Among MLWH diagnosed with HIV infection before immigration, 361/652 (55%) were men and the median age was 33 (IQR: 28–40) years. Overall, SSA was the predominant region of origin (Table 1), but over time, Western countries became the predominant region of origin (Table 2). The proportion of men also increased over time. The demographic characteristics for each region resembled those of MLWH diagnosed after immigration, as described above. The median CD4^+^ T-cell count increased from 270 (IQR: 133–488) cells/µL in 1995–99 to 600 (IQR: 446–774) cells/µL in 2015–20. The number of MLWH presenting to Danish HIV care with a CD4^+^ T-cell count < 350 cells/µL or AIDS decreased from 44 (59%) in 1995–99 to 19 (13%) in 2015–20. Likewise, the proportion of MLWH presenting with VL < 200 copies/ml increased from 11% in 1995–99 to 83% in 2015–20.

#### Time from immigration to LTC

The overall median time from immigration to LTC was 38 (IQR: 0–105) days, ranging from 5 (IQR: 0–74) days for MLWH from SSA to 62 (IQR: 22–169) days for MLWH from Western countries (Figure 1). A sensitivity analysis excluding the 212 MLWH with LTC in Denmark occurring before official immigration changed the estimate to 75 days (IQR: 35–186). The proportion of MLWH with LTC within 90 days of immigration ranged from 79% (SSA) to 62% (Western countries). Time from immigration to LTC increased from 2000 to 2004 and onward (Figure 3).

## Discussion

In this nationwide study, we demonstrated that MLWH had resided a median of 3.7 years in Denmark before diagnosis of HIV infection with no change over time from 1995 to 2020. However, regardless of whether migrants were diagnosed with HIV infection in Denmark or arrived in Denmark with a known HIV diagnosis, linkage to care was timely for a high (62%–95%) proportion of MLWH. Consistent with other studies [9,26], we found high proportions of LP and AHD among MLWH diagnosed after immigration. In comparison, the proportion of LP among Danish-born individuals has been estimated to be 42% [9].

Although the median time between immigration and diagnosis remained unchanged, the proportions of LP, AHD and AIDS at time of LTC decreased in later calendar periods. Consistent with this, the CD4^+^ T-cell counts at LTC were higher in the later calendar periods. These changes mainly reflect that the composition of the migrant population changed over time, with fewer migrants from SSA following increasingly restrictive migration policies in Denmark [27]. Furthermore, the global HIV pandemic has changed following the increased focus on early and regular HIV testing and immediate ART provision. The proportion of MLWH – especially from Western countries – who were already diagnosed with HIV and on effective ART at time of immigration increased over time. This might explain why time from immigration to LTC increased over time, as MLWH already on ART at immigration might bring medicine and have no need of immediate healthcare.

In line with other studies [9], our findings illustrate that MLWH constitute a heterogenous population with different trends across geographical regions of origin. In addition, the interpretation and clinical implications of measuring time from immigration to HIV diagnosis are different for subpopulations of MLWH. We presume that MLWH from the high prevalence regions of SSA and East and South Asia were mainly infected with HIV before immigration to Denmark [7,9,20] and, consequently, most of the time spent in Denmark before HIV diagnosis reflected a diagnostic delay. In both subpopulations, we found consistently high proportions of LP and AHD. This makes it crucial to reduce the diagnostic delays, which even during 2015–20 exceeded 2 years for MLWH from SSA and 4 years for MLWH from East and South Asia. Other studies have also identified migrants from SSA and East and South Asia to be at high risk of late presentation [2,8,9], and modelling studies have identified risk of delays in the HIV continuum of care for these migrant subpopulations [20,28]. We saw a high correlation between geographic region of origin and sex. MLWH from SSA and East and South Asia were more likely to be women, and in line with this, we found high proportions of LP and ADH among women.

Compared with migrants from SSA and East and South, MLWH from Western countries are more likely to become infected with HIV after immigration, especially among MSM [7]. Thus, for migrants from Western countries, the observed large dispersion and sometimes long time periods between immigration and HIV diagnosis probably represent a combination of diagnostic delays and post-migration HIV transmission. As a consequence, healthcare efforts towards this migrant subpopulation should focus on prevention of new infections [7] in addition to continuous HIV testing efforts.

MLWH from eastern Europe constituted an increasing proportion of the total population of MLWH over calendar time. Eastern Europe is one of the few regions where the HIV epidemic is growing with a high incidence:prevalence ratio of 10% [29]. Our results underline that the risk of HIV infection among key populations in eastern Europe should be targeted not only in the region but also recognised among migrants from the region.

Over time, the proportion of MSM MLWH increased. This reinforces that HIV prevention and testing efforts should ensure initiatives aimed at MSM migrants.

Healthcare is free of charge in Denmark, and healthcare efforts have been very effective in reducing the HIV epidemic among Danish-born MSM [18,19,30]. Nevertheless, we still face a problem with migrants’ access to HIV testing. It is essential to make anonymous, voluntary HIV testing available free of charge to everyone, both at a community level and at primary healthcare providers, and to ensure provider-initiated (voluntary) testing, so that migrants who do not perceive themselves at risk of HIV are still offered tests. However, several studies have shown how structural barriers prevent migrants from seeking timely HIV diagnosis and care [10-12]. Furthermore, a recent study has pointed to many missed opportunities for provider-initiated HIV testing at contact with the primary healthcare system for people later diagnosed with HIV in Denmark [31]. Our study demonstrates that the barriers to HIV testing in combination with missed testing opportunities potentially cause diagnostic delays of several years.

Other studies have also shown how migrants are at increased risk of late presentation [2,8,9], our study further elucidates that while some MLWH arrive as LPs, others likely become LPs after immigration because of diagnostic delays. This implies that increased and diverse HIV testing efforts in the migrants’ country of arrival, combined with the breakdown of barriers, have the potential to reduce morbidity and mortality associated with presentation with advanced HIV disease. 

This population-based study provides new insights into the extend of the delays in the first steps in the HIV continuum of care among migrants. The strengths of our study include the population-based and nationwide design. In contrast to another Danish study, which only included refugees and family-reunified immigrants [9], our study included all migrants with residence in the country. The use of a population-based clinical database linked to Danish health and administrative registers allowed us to trace patients retrospectively from date of LTC without recall bias. Although this work was planned as a descriptive study and we did not include a multivariate analysis, the large population enabled us to stratify our analysis according to region of origin, calendar period and late presentation. 

Our study had some limitations. The study design and number of migrants did not allow us to take socioeconomic differences within regions and migrant status into account. CRS registers arrival in Denmark as the date of obtaining official residence, which means we were unable to study undocumented migrants living in Denmark without residence and with limited access to healthcare. This may have led us to underestimate the true delay in HIV diagnosis among MLWH. We were unable to take post-migration HIV transmission fully into account, and the assumption that migrants from high prevalence regions are mostly HIV-infected before immigration may not always be correct [32]. Our study design only allowed us to include MLWH once they were linked to care. The number of MLWH diagnosed with HIV but never linked to care is most likely very small. It is mandatory to report HIV infections, and HIV care is very structured in Denmark. However, presumably, a number of MLWH with short stays in Denmark have maintained contact with the healthcare system in their home country, and consequently their HIV status would not be registered in Danish national registries.

## Conclusion

This nationwide study of MLWH in Denmark demonstrates that, while almost every aspect of HIV healthcare has improved greatly over the last decades, we still face the challenge that many MLWH, including those with late presentation and presentation with advanced HIV disease, have resided years in Denmark before being diagnosed with HIV infection. These results point to missed opportunities for both detection and reducing late presentation among MLWH in Denmark. Our findings call for an increased effort to systematically implement voluntary HIV testing, and to make it more accessible for migrants – especially for those arriving in Denmark from high HIV prevalence areas and for women. Work should be done to investigate and combat the barriers preventing migrants from seeking timely HIV testing, to close this crucial gap in the HIV continuum of care.

## Supplementary Data

153000 Supplement
