# Supplementary material for: Length of stay in Denmark before HIV diagnosis and linkage to care: a population-based study of migrants living with HIV, Denmark, 1995 to 2020
Source: Euro Surveill. 2022 Jul 28;27(30):2100809. doi: 10.2807/1560-7917.ES.2022.27.30.2100809 (PMC9336168; doi:10.2807/1560-7917.ES.2022.27.30.2100809)

Supplementary material

This supplementary material is hosted by *Eurosurveillance* as supporting information alongside the article *Length of stay before HIV diagnosis and linkage to care: a population-based study of migrants, Denmark, 1995 to -2020* on behalf of the authors who remain responsible for the accuracy and appropriateness of the content. The same standards for ethics, copyright, attributions and permissions as for the article apply. Supplements are not edited by Eurosurveillance and the journal is not responsible for the maintenance of any links or email addresses provided therein.

Table of contents

Appendix 1. Country codes

Appendix 2: Supplementary tables

Appendix 3: Supplementary figures

Country codes

Sub-Saharan Africa:

Angola, Benin, Botswana, Burkina Faso, Burundi, Cameroun, Central African Republic, Congo (DR), Congo (Republic), Cote d'Ivoire, Djibouti, Eswatini, Ethiopia, Eritrea, Gabon, Gambia, Ghana, Guinea, Guinea Bissau, , Kenya, Lesotho, Liberia, Madagascar, Malawi, Mauritania, Mauritius, Mozambique, Namibia, Niger, Nigeria, Rwanda, Sierra Leone, Somalia, South Africa, South Sudan, Tanzania, Togo, Uganda, Zimbabwe, Zambia

Western countries:

Australia, Austria, Belgium, Bosnia and Herzegovina, Canada, Croatia, Faroe Islands, Finland, France, Germany, Great Britain, Greece, Iceland, Ireland, Italy, Netherlands, New Zealand, North Macedonia, Norway, Portugal, Serbia, Slovenia, Spain, Sweden, Switzerland, USA, Yugoslavia

East and South Asia:

Afghanistan, Bangladesh, Cambodia, China, India, Indonesia, Iran, Japan, Korea, Malaysia, Myanmar, Nepal, Pakistan, the Philippines, Singapore, Sri Lanka, Taiwan, Thailand, Vietnam

Eastern Europe:

Belarus, Bulgaria, Czech Republic, Estonia, Hungary, Latvia, Lithuania Poland, Romania, Russia, Slovakia, Turkmenistan, Ukraine, Uzbekistan

Other:

Algeria, Argentina, Armenia, British Virgin Islands, Bolivia, Brazil, Chile, Colombia, Costa Rica, Cuba, Cyprus, Dominican Republic, Ecuador, Egypt, El Salvador, Georgia, Guatemala, Guyana, Haiti, Honduras, Iraq, Israel, Jamaica, Jordan, Kuwait, Lebanon, Libya, Mexico, Morocco, Panama, Peru, Saudi Arabia, Sudan, Syria, Trinidad and Tobago, Tunisia, Turkey, Uruguay, Venezuela, Yemen

Supplementary table S1: Late presentation and presentation with advanced HIV disease among migrants from Sub-Saharan Africa and East and South Asia stratified by calendar periods

Calendar Period	1995-1999	2000-2004	2005-2009	2010-2014	2015-2000
**Sub-Saharan Africa**					
Late Presenters, n (%*)	106 (69)	122 (74)	76 (63)	70 (66)	42 (60)
Advanced HIV disease, n (%*)	67 (44)	88 (53)	37 (31)	47 (44)	25 (36)
**East and South Asia**					
Late Presenters, n (%*)	33 (73)	38 (62)	40 (59)	34 (59)	36 (71)
Advanced HIV disease, n (%*)	18 (40)	31 (51)	26 (38)	26 (45)	22 (43)

*Percentage of total number of migrants with CD4 counts from the region in the calendar period

Supplementary figure S1: Years (median, IQR) from immigration to HIV-diagnosis for MLWH from Sub-Saharan Africa and East and South Asia stratified by calendar period

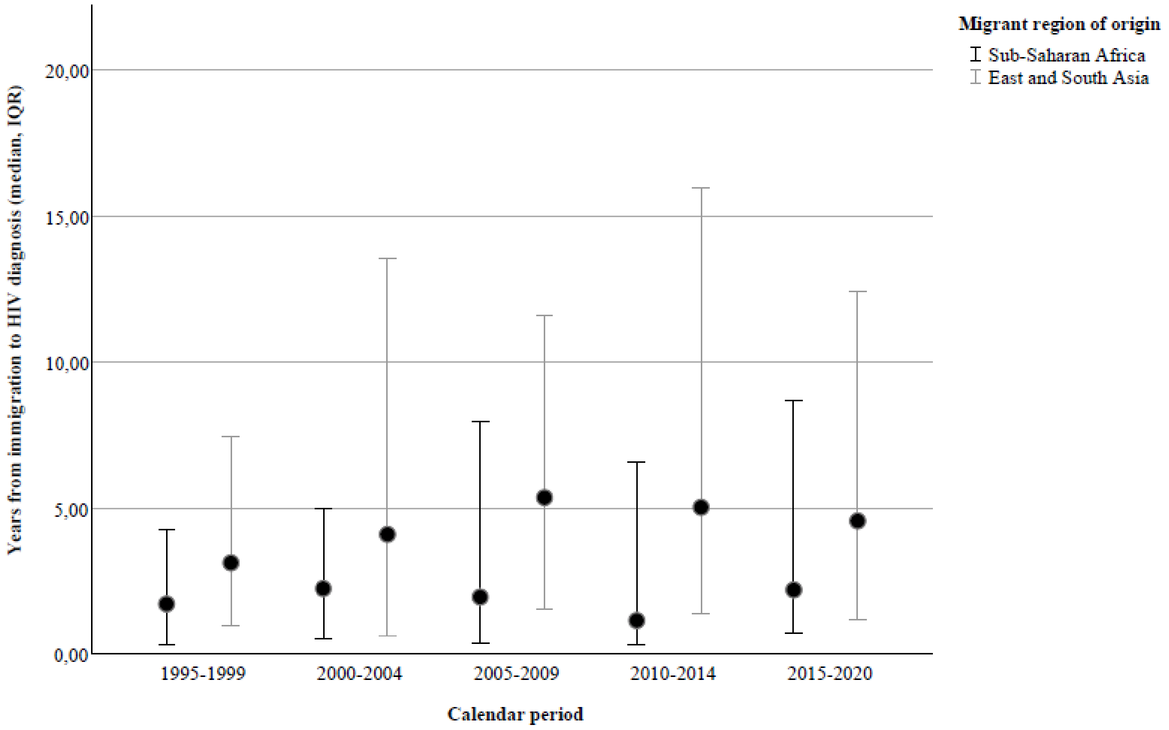

Supplementary figure S2: Years (median, IQR) from immigration to HIV-diagnosis for late presenters, stratified by calendar period

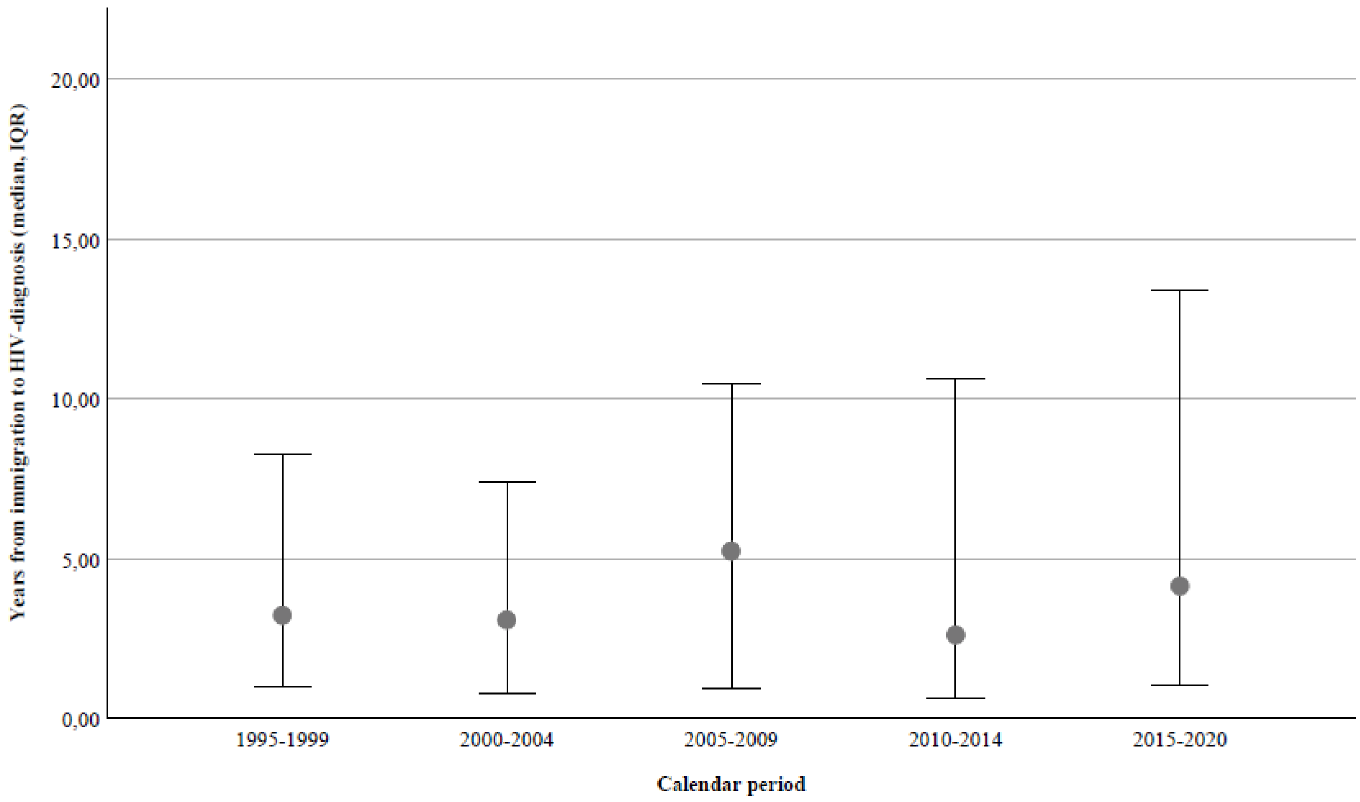

Supplement: Supplement [file 21-00809_BORCHMANN_Supplement.pdf]
